# Ataxin-3, The Spinocerebellar Ataxia Type 3 Neurodegenerative Disorder Protein, Affects Mast Cell Functions

**DOI:** 10.3389/fimmu.2022.870966

**Published:** 2022-04-26

**Authors:** Anna S. Sowa, Eva Haas, Jeannette Hübener-Schmid, Axel Lorentz

**Affiliations:** ^1^ Institute of Nutritional Medicine, University of Hohenheim, Stuttgart, Germany; ^2^ Institute of Medical Genetics and Applied Genomics, University of Tübingen, Tübingen, Germany

**Keywords:** ataxin-3, MJD, SCA3, mast cell, cytokine, neurodegeneration, inflammation

## Abstract

Spinocerebellar ataxia type 3 (SCA3), also known as Machado-Joseph Disease, is a progressive neurodegenerative disorder characterized by loss of neuronal matter due to the expansion of the CAG repeat in the *ATXN3/MJD1* gene and subsequent ataxin-3 protein. Although the underlying pathogenic protein expansion has been known for more than 20 years, the complexity of its effects is still under exploration. The ataxin-3 protein in its expanded form is known to aggregate and disrupt cellular processes in neuronal tissue but the role of the protein on populations of immune cells is unknown. Recently, mast cells have emerged as potential key players in neuroinflammation and neurodegeneration. Here, we examined the mast cell-related effects of ataxin-3 expansion in the brain tissues of 304Q ataxin-3 knock-in mice and SCA3 patients. We also established cultures of mast cells from the 304Q knock-in mice and examined the effects of 304Q ataxin-3 knock-in on the immune responses of these cells and on markers involved in mast cell growth, development and function. Specifically, our results point to a role for expanded ataxin-3 in suppression of mast cell marker CD117/c-Kit, pro-inflammatory cytokine TNF-α and NF-κB inhibitor IκBα along with an increased expression of the granulocyte-attracting chemokine CXCL1. These results are the beginning of a more holistic understanding of ataxin-3 and could point to the development of novel therapeutic targets which act on inflammation to mitigate symptoms of SCA3.

## Introduction

Spinocerebellar ataxia type 3 (SCA3), also known as Machado-Joseph disease (MJD), belongs to the group of neurodegenerative disorders known as polyglutamine (polyQ) disease associated with a polyQ expansion in the protein expressed by the corresponding causative gene (1). In SCA3, the polyQ-expanded protein is known as ataxin-3 and patients carry an expansion between 61 and 87 repeats on the affected allele resulting in the observed phenotype characterized by a disturbance of movement coordination (cerebellar ataxia), bulbar, pyramidal and extrapyramidal signs, and a possible occurrence of peripheral neuropathy or ophthalmoplegia (2). Currently, there is no cure or no therapy mitigating disease progression available for SCA3.

Although in the 20 years since the discovery of the ataxin-3 protein large strides have been made to understand the cellular dysfunction underlying the disease such as, mitochondrial and autophagic disturbances, as well as the protein cleavage and aggregation (3), the full mechanisms behind neuronal death are still unknown. The newly developed 304Q ataxin-3 knock-in mouse model is an important promising tool to further reveal the pathologic effects of ataxin-3 expansion (4). These knock-in mice express 304Q instead of the 6Q normally present in either one or both of the murine ataxin-3 alleles. With twelve months of age these heterozygous and homozygous mice show a significant weight reduction compared to wild type (WT) mice. Despite a very mild motor phenotype, they already show massive SDS-insoluble ataxin-3 and ubiquitin-positive aggregates throughout the brain supporting a strong positive correlation between the polyQ expansion and a toxic function of ataxin-3 related to neurons (4).

Mast cells are important players in the immune system and exert their effects *via* expression and release of mediators involved in physiological functions of the body including innate and adaptive immune response, tissue healing, angiogenesis, and normal neuronal growth (5). The best-known activation of mast cells occurs during type I allergic reactions through crosslinking of immunoglobulin (Ig) E bound FcϵRI receptors by antigens. Degranulation occurs a few seconds after crosslinking and results in release of granules stored mediators attracting leukocytes such as eosinophils, neutrophils, or Th2 lymphocytes and increasing permeability of the blood vessels to allow immune cell passage (5, 6). In addition to the FcϵRI, mast cells express a variety of receptors such as other Fc receptors, receptors for chemokines and cytokines, or receptors for pathogen associated molecular patterns such as Toll-like receptors, all involved in mast cell activation and immune response (5, 6). In the last decade, mast cells have gained traction in their role in neurodegeneration. They are incredibly potent and considered the first responders able to initiate and magnify immune responses in the brain partly due to their capacity to access neuronal tissue and to affect the blood brain barrier (7). Although mast cells are important immune cells, their role in SCA3 remains to be discovered. It is still unclear if mast cells could play a pathogenic role as mediators of ataxin-3 activity.

The role of ataxin-3 in cell autonomous inflammatory pathways is only beginning to be examined (8). Therefore, we analyzed the effects of ataxin-3 expansion in the brain tissues of the 304Q ataxin-3 knock-in mice and the cerebellar post-mortem brain tissue of SCA3 patients on markers involved in mast cell growth, development and function. For these studies, we also established cultures of mast cells from the 304Q ataxin-3 knock-in mice. Our results point to a role for expanded ataxin-3 in suppression of mast cell marker CD117/c-Kit, pro-inflammatory cytokine TNF-α and NF-κB inhibitor IκBα along with an increased expression of the granulocyte-attracting chemokine CXCL1. This is the first report to describe an immune cell phenotype for SCA3 and a mast cell-specific phenotype for neurodegenerative conditions.

## Materials and Methods

### Generation of 304Q Ataxin-3 Knock-In Mice

This study was carried out in strict accordance with the recommendations presented in the Guide for Care and Use of Laboratory Animals of the University of Tübingen, Germany. The protocols were approved by the Institutional Animal Care and Use Committee (IACUC) of the University of Tübingen, Germany. Ataxin-3 knock-in C57Bl/6 mice with 304Q were generated using zinc finger technologies and providing a donor vector with (CAACAGCAG)48 as described (4). One out of three founders integrated up to 304 CAGs into the *ataxin-3* gene. 304Q ataxin-3 knock-in mice used in the current study were either heterozygous or homozygous. Mouse housing and genotyping were described earlier (4). For qRT-PCR 3 mice per genotype at the age of 2 and 12 months and western blot analyses 4 mice per genotype were analyzed at the age of 3 and 12 months.

### Histological Analysis of Tissue Mast Cells

Formalin-fixed tissue samples were embedded in paraffin. After deparaffinization and rehydration, 5 µM thick sections were stained with toluidine blue (Carl Roth, Karlsruhe, Germany) for visualization of mast cells. Mast cells were counted at 200-400x magnification. Microscopic analysis was performed using AxioVision software (Carl Zeiss Microscopy, Jena, Germany).

### Generation and Culture of Bone Marrow-Derived Mast Cells (BMMC)

Bone marrow cells were collected from femurs of homozygous (304Q/304Q) and heterozygous (WT/304Q) 12 month old 304Q ataxin-3 knock-in and wild type (WT/WT) mice and cultured in RPMI (Gibco, Thermo Fisher Scientific, Waltham, MA), supplemented with 10% fetal bovine serum, penicillin/streptomycin (Biochrom, Berlin, Germany), plus 30 ng/mL mouse IL-3 (PeproTech, Hamburg, Germany). Cells were cultured in 6 well plates, incubated at 37°C in a humidified incubator under 5% (v/v) CO2. Starting after 3 days, half of the medium was changed once a week every week. In addition, starting after 3 weeks, medium was changed completely once a week every week by transferring the non-adherent cells in fresh medium into a new plate. After culture for 9 weeks, cells were used for functional assays. For each genotype, several lines were generated. Maturity and purity of the BMMC were examined on cytospins stained with May-Grünwald/Giemsa (Carl Roth). We did not detect differences in the staining of pure BMMC from 304Q/304Q mice compared to BMMC from WT/WT mice (not shown).

### Treatment and Analyses of BMMC

To detect release of β-hexosaminidase, BMMC were loaded with 2,4-dinitrophenyl (DNP)-specific IgE for 90 min and stimulated with 0.1 µg/ml DNP (Thermo Fisher Scientific), for 90 min. To analyze phosphorylation status of signaling molecules, BMMC were stimulated with DNP/IgE or with 1 µM phorbol ester PMA and 1 µM ionomycin (*In vivo*Gen, San Diego, USA) for 10 min or with 1 μg/ml lipopolysaccharide (LPS) from Escherichia coli 0111:B4 (Sigma-Aldrich, Munich, Germany) for 30 min. To analyze cytokine expression and release, BMMC were stimulated for 90 min or for 6 h and culture supernatants were tested by ELISA (R&D systems, MN) in accordance with the manufacturer’s instructions. Degranulation of BMMC was quantified by determining the amount of released β-hexosaminidase in the supernatant by color enzyme assay (9). The percent degranulation of mast cells was estimated by the following formula: (β-hexosaminidase activity in the supernatant fraction/total β-hexosaminidase activity in the cellular and supernatant fraction) ×100.

### RNA Preparation and Real-Time RT-PCR

For analysis of BMMC, total RNA was prepared by using EXTRACTME RNA isolation kit (7Bioscience, Hartheim, Germany). Real-time RT-PCR was performed using SsoFastTM EVAGreen Supermix (Bio Rad Laboratories, Munich, Germany) and as described (10). To analyze data, a relative quantification method was performed by using the housekeeping gene of glyceraldehyde 3-phosphate dehydrogenase (*Gapdh*) or *β-actin* as a reference for expression of the target gene. The CFX Manager 2.1 software of BioRad Laboratories was used. For analysis of human and murine brain tissue, total RNA was prepared using AllPrep DNA/RNA/microRNA Universal Kit (Qiagen, Hilden, Germany) as described earlier (4). 500 ng purified RNA was transcribed into cDNA using QuantiTect Reverse Transcription Kit (Qiagen) Real-time RT-PCR was performed using SYBR Green PCR Master Mix (Qiagen) as described. qRT-PCR was run on the LightCycler 480 II (Roche, Mannheim, Germany). Relative gene expression quantification was performed by normalization to the housekeeping genes *Sdha*, *Pdh*, *mActb* and *Tbp* using LightCycler 480 SW 1.5.1 software (Roche). Specific sense/anti-sense primers: human *ACTB*: 5’ AAA GAC CTG TAC GCC AAC AC/5’ CTC AGG AGG AGC AAT GAT CT, human *CD117*: 5’ GCC CAA TAT AAA AGG CAA AT/5’ AGT GCA AAT GGT TAC TTC CA, human *IL-3RA*: 5’ CCT CCT TTG GCT CAC GCT G/5’ GCC CAC TCG GAC GGT GTA G, human *TNF*: 5’ CAG ATA GAT GGG CTC ATA CCA GGG/5’ GCC CTC TGG CCC AGG CAG TCA G, human *CXCL8*: 5’ CTG AGA GTG ATT GAG AGT GG/5’ ACA ACC CTC TGC ACC CAG TT, mouse *Gapdh*: 5’ TGT TCC TAC CCC CAA TGT GT/5’ AGA GTG GGA GTT GCT GTT GA, mouse *Cd117*: 5’ CTG GTG GTT CAG AGT TCC ATA GAC/5’ TCA ACG ACC TTC CCG AAG G, mouse *Il-3rα chain*: 5’ TGG AGG AAG TCG CTG CTC TA/5’ CGT CAC CTC GCA GTC TTC AA, mouse *Tnf*: 5’ GGA GGC AAC AAG GTA GAG/5’ TGT CCA TTC CTG AGT TCT G, mouse *Cxcl1*: 5’ TAG GGT GAG GAC ATG TGT G/5’ GCC CTA CCA ACT AGA CAC AA, mouse *Sdha*: 5’ GCA GCA CAG GGA GGT ATC A/5’ CTC AAC CAC AGA GGC AGG A, mouse *Pdh*: 5’ GTA GAG GAC ACG GGC AAG AT/5’ TGA AAA CGC CTC TTC AGC A, mouse *mActb*: 5’ CCA CAC CCG CCA CCA GTT CG/5’ TAC AGC CCG GGG AGC ATC GT, mouse *Tbp*: 5’ TCT ATT TTG GAA GAG CAA CAA AGA C/5’ GAG GCT GCT GCA GTT GCT A

### Western Blot Analysis

Western blot analysis of BMMC was performed as described previously (11) and murine brain lysates as described in Haas et al. (4). Shortly, frozen brain tissue was homogenized in TES buffer (4% Tris Base pH 7.5, 0.1 mM EDTA, 100 mM Na2Cl) containing protease inhibitor complete EDTA-free protease inhibitor cocktail (Roche). Afterwards, TNES (TES-buffer plus 10% Igepal CA-630) was added in a relation of 1:10 and homogenates incubated on ice for 30 min. Homogenates were centrifuged at 13.200 g for 25 min at 4°C and to supernatants glycerol to a final concentration of 10% were added. For Western blot analyses, 4x LDS sample buffer (1 M Tris base pH 8.5, 2 mM EDTA, 8% LDS, 40% glycerol, 0.025% phenol red) and 100 mM 1,4-dithiothreitol (MerckMillipore) was added to 30 µg of protein lysates and heat-denatured at 70°C for 10 min. Protein samples were separated electrophoretically using 8% Bis-Tris gel and MOPS (50 mM MOPS, 50 mM Tris base pH 7.3, 3.5 mM SDS, 1 mM EDTA) electrophoresis buffer. Proteins were transferred on 0.2 um nitrocellulose membrane (GE Healthcare) using a Bicine/Bis-Tris transfer buffer (25 mM Bicine, 25 mM Bis-Tris pH 7.2, 1 mM EDTA, 15% methanol). After membrane blocking with 5% skimmed milk powder (Sigma-Aldrich), membranes were incubated with the respective primary antibody at 4°C overnight. For visualization of proteins of brain lysates, membranes were incubated with fluorescence tagged secondary antibodies (IRdye 800CW goat anti-mouse IgG (H+L), LI-COR or IRdye 800CW goat anti-rabbit IgG (H+L) LI-COR) for 1.5 hours at room temperature and fluorescence signal detection was performed on the LI-COR ODYSSEY FC (LI-COR). Visualization of proteins of BMMC was performed by the secondary antibodies anti-mouse IgG or anti-rabbit IgG HRP-linked (Cell Signaling Technology^®^) and Super Signal^®^ West Dura Extended Duration Substrate (Thermo Fisher Scientific, Waltham, MA). The obtained signals were measured by bioimaging analyzer (Alpha Innotech Corporation, San Leandro, CA). The membranes were stripped and used for a second or third staining. Membranes were probed with phospho-ERK (MAPK)-1/2 (12D4) mouse mAb, IκBα (44D4) rabbit mAb, CD117 (D13A2) rabbit mAb (all from Cell Signaling Technology^®^, Frankfurt, Germany), TNF alpha (EPR19147) rabbit mAb (from Abcam), β-Actin (AC-15) mouse mAb (Sigma Aldrich), GAPDH (D-6) mouse mAb (from Santa Cruz Biotechnology, Dallas, TX). β-Actin or GAPDH labeling was used as loading control for normalization.

### Statistics

Data expressed as mean ± SEM. Unpaired t-test was used to analyze differences between two groups. Two-way analysis of variance was used to analyze differences between genotypes and stimulation conditions with Bonferroni multiple comparisons. A value of p < 0.05 was considered to be statistically significant.

## Results

### Brain Tissues of 304Q Ataxin-3 Knock-In Mice and Post-Mortem SCA3 Patients Show an Effect of Expanded Ataxin-3 on the Expression of Mast Cell Markers and Inflammatory Cytokines

**Figure 1:**
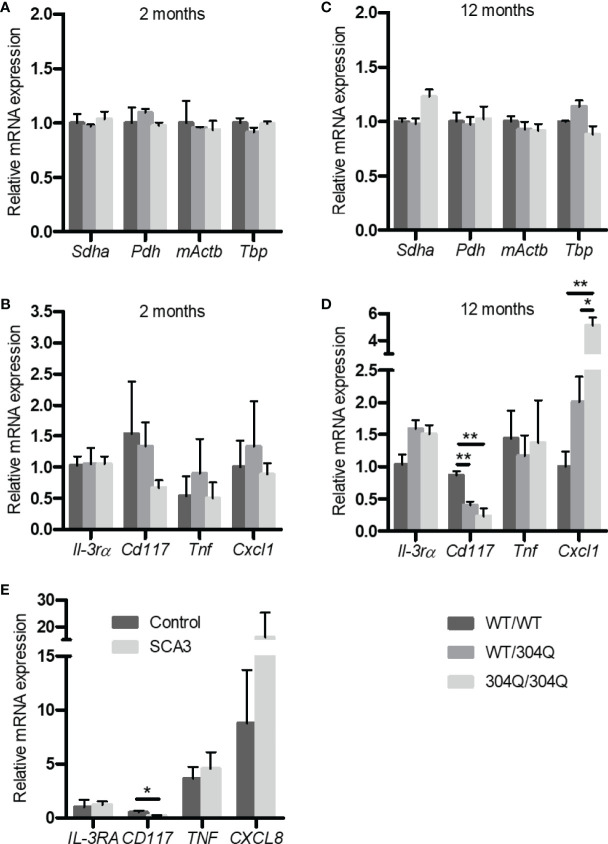
Expression of *Il-3rα*, *Cd117*, *Tnf*, and *Cxcl1*/*mKc* as well as the housekeeping genes *Sdha*, *Pdh*, *mActb* and *Tbp* mRNA in brain tissue from 2-month-old **(A, B)** and from 12-month-old **(C, D)** WT/WT, WT/304Q, and 304Q/304Q ataxin-3 knock-in mice. Unpaired t test shows statistically significant differences, n = 3, Values are mean ± SEM, *p < 0.05, **p < 0.01. Expression of *CD117*, *IL-3RA*, *TNF*, and *CXCL8* mRNA in cerebellar brain tissue of SCA3 patients normalized using *β-actin* **(E)**. Total RNA was extracted, reverse-transcribed and analyzed by quantitative real-time PCR, n = 4-6. Values are mean ± SEM, p values were calculated by unpaired t-test, *p < 0.05.

In order to elucidate the effects of polyglutamine expansion of ataxin-3 on mast cells we chose to analyze the mRNA expression levels of the mast cell markers CD117/c-Kit and IL-3 receptor α-chain (IL-3Rα) using 304Q ataxin-3 knock-in mice. The 304Q ataxin-3 knock-in mouse model represents the human patient phenotype on a neuropathological, behavioral, and transcriptomic levels in a more complete manner than any existing SCA3 mouse. CD117 is a mast/stem cell receptor for growth factor SCF important in the mast cell development and function, while IL-3R is a potent receptor involved in the stimulation of mast cell growth in response to IL-3 produced by activated T cells (12). There was both a significant down-regulation of *Cd117* expression and a significant up-regulation of *mKc/Cxcl1* expression in the brains of 12-month-old 304Q/304Q and WT/304Q mice compared to WT/WT mice (**Figures 1B, D**). Also, the brains of 304Q ataxin-3 knock-in mice were tested for expression of the cytokine gene *Tnf* and the chemokine *mKc/Cxcl1*. *Tnf* is well known to participate in an acute inflammation and *mKc/Cxcl1* (homologue to human *IL-8/CXCL8*) is involved in pro-inflammatory chemotaxis of mast cells. The *Cxcl1* expression was reliably increased, but no changes in the levels of *Tnf* expression were detected in 304Q ataxin-3 knock-in mice relative to WT/WT mice. The housekeeping genes *Sdha*, *Pdh*, *mActb*, and *Tbp* were not affected (**Figures 1A, C**).

In order to demonstrate the relevance of our findings in the SCA3 mouse model to human disease, we tested the expression of *IL3RA*, *TNF* and *CXCL8* in the post-mortem brain tissues of human SCA3 patients. Noteworthy, we found a tendency towards an increased expression of *CXCL8* associated with a significantly decreased expression of *CD117* compared to controls consistent with our observations in 304Q ataxin-3 knock-in mice (**Figure 1E**).

**Figure 2:**
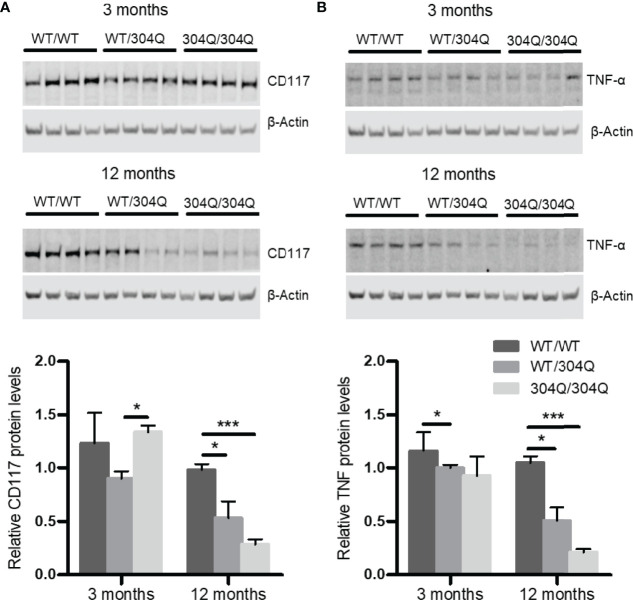
CD117 and TNF-α are down-regulated in cerebellar brain tissue of 304Q/304Q ataxin-3 knock-in mice. Protein levels of CD117 **(A)** and TNF-α **(B)** in relation to β-actin in brain tissue from 3-month-old and from 12-month-old WT/WT, WT/304Q, or 304Q/304Q ataxin-3 knock-in mice. Unpaired t test shows statistically significant differences, n = 4. Values are mean ± SEM, *p < 0.05, ***p < 0.001.

To confirm the findings for gene expression on protein level, we analyzed the expression of CD117 and TNF-α in brain tissues by Western blot. A significant down-regulation of both CD117 and TNF-α was detected in the 12-month-old 304Q/304Q and WT/304Q mice compared to WT/WT mice (**Figure 2**). We have not established mast cell staining in the available brain tissues, but we have stained mast cells in intestinal tissues. There was a trend towards lower mast cell numbers in duodenal and jejunal tissues of 304Q/304Q ataxin-3 knock-in mice compared to WT controls (**Supplementary Figure S1**).

### PolyQ-Expanded Ataxin-3 Modulates Expression of CD117 and Cytokines in BMMC

Next, we aimed to analyze the effect of ataxin-3 directly in mast cells. Thus, we generated cultures of BMMC from the 12-month-old WT/WT, WT/304Q, or 304Q/304Q ataxin-3 knock-in mice. Interestingly, culturing bone marrow cells from 304Q/304Q ataxin-3 knock-in the presence of IL-3 for 6 weeks resulted in 71 ± 9.5% mature BMMC compared to 91 ± 1.3 and 97 ± 2% from WT/WT or WT/304Q mice, respectively. Pure BMMC were found in all cases following 9 weeks of culture. Recovery was 406 ± 159% of BMMC from WT/WT mice compared to 234 ± 33% and 225 ± 82% of BMMC from WT/304Q or 304Q/304Q ataxin-3 knock-in mice. No differences concerning size and degree of granulation were observed between mature BMMC derived from WT/WT, WT/304Q, or 304Q/304Q ataxin-3 knock-in mice.

**Figure 3:**
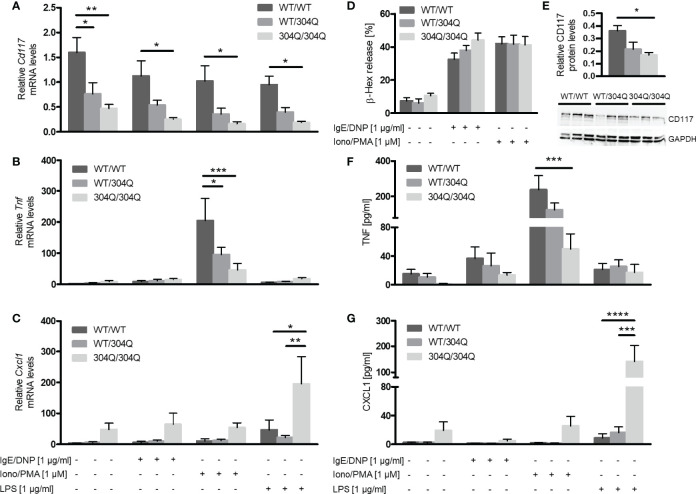
Expression of *Cd117* and *Tnf* is down-regulated and expression of *Cxcl1* is upregulated in BMMC from 304Q/304Q ataxin-3 knock-in mice. *Cd117* **(A)**, *Tnf* ***(*B*)***, and *Cxcl1* **(C)** mRNA levels were determined following stimulation of BMMC from WT/WT, WT/304Q, or 304Q/304Q ataxin-3 knock-in mice with IgE/DNP, ionomycin/PMA or LPS for 90 min. Release of β-hexosaminidase (β-Hex) **(D)** as a marker of degranulation was measured following stimulation with IgE/DNP or ionomycin/PMA. Protein levels of CD117 in relation to GAPDH were measured in BMMC from WT/WT, WT/304Q, or 304Q/304Q ataxin-3 knock-in mice **(E)**. Release of cytokines TNF-α **(F)** and CXCL1 **(G)** in supernatants of BMMC from 304Q ataxin-3 knock-in mice were measured following stimulation with IgE/DNP, ionomycin/PMA or LPS for 6h Unpaired t test **(E)** and two-way ANOVA **(A–D, F, G)** show statistically significant differences, n = 3-5. Values are mean ± SEM, *p < 0.05, **p < 0.01, ***p < 0.001, ****p < 0.0001.

Expression levels of *Cd117*, *Tnf* and *Cxcl1* mRNA were determined before and after stimulation of BMMC with IgE/2,4-dinitrophenyl (DNP), ionomycin/phorbol-12-myristat-acetate (PMA) or lipopolysaccharide (LPS). The *Cd117* expression was strongly reduced in BMMC from homozygous and, to a lesser extent, heterozygous 304Q ataxin-3 knock-in mice compared to cells from WT/WT mice (**Figure 3A**). This finding could be confirmed on protein level by Western blot, which showed a significantly lower expression of CD117 in BMMC from 304Q/304Q ataxin-3 knock-in compared to cells from WT/WT mice (**Figure 3E**). *Tnf* expression in response to ionomycin/PMA stimulation was substantially reduced in cells from 304Q/304Q and WT/304Q mice compared to WT/WT controls (**Figure 3B**). In contrast, a marked increase of mRNA expression for the proinflammatory chemokine gene *Cxcl1* was detected in BMMC from 304Q/304Q mice relative to WT/WT mice in response to LPS stimulation (**Figure 3C**). These observations are in accordance with our findings in mouse and human SCA3 brain tissues.

### 304Q Ataxin-3 Modulates Cytokine Release by BMMC

BMMC from 12-month-old 304Q ataxin-3 knock-in mice were stimulated with either IgE/DNP or ionomycin/PMA for 90 min and the release of pre-stored β-hexosaminidase as marker of degranulation was measured (**Figure 3D**). In both cases the stimulated BMMC demonstrated robust degranulation; however, there was no difference between the responses of BMMC from 304Q ataxin-3 knock-in mice compared to WT/WT mice demonstrating that the expanded 304Q ataxin-3 did not influence the capacity of BMMC to degranulate (**Figure 3D**).

To analyze the release of *de novo* synthesized cytokines, BMMC from 12-month-old 304Q ataxin-3 knock-in mice were stimulated with either IgE/DNP, ionomycin/PMA, or LPS for 6 h. The amount of released TNF-α protein measured by ELISA showed a strong decrease upon ionomycin/PMA stimulation in BMMC from 304Q/304Q mice compared to WT/WT mice (**Figure 3F**) in agreement with the gene expression data. The release of CXCL1 also correlated to the measured mRNA levels, with a strong increase in CXCL1 levels in BMMC from 304Q/304Q mice (**Figure 3G**).

### 304Q Ataxin-3 Modulates Activation of NF-κB Signaling Pathway in BMMC

Further, we aimed to analyze the signaling pathways altered in BMMC from 304Q ataxin-3 knock-in 3 mice compared to BMMC from WT/WT mice to observe which proteins could be responsible for the transcriptional changes in cytokine production. We analyzed two main signaling pathways involved in mast cell cytokine expression, the MAPK pathway ERK1/2 and the NF-κB pathway.

**Figure 4:**
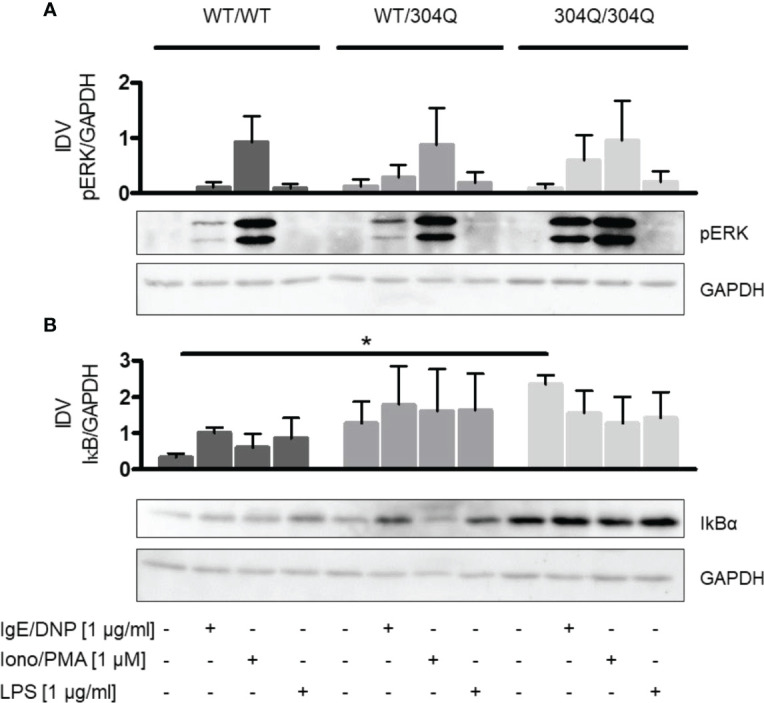
ERK1/2 and NF-κB signaling in BMMC from WT/WT, WT/304Q, or 304Q/304Q ataxin-3 knock-in mice. **(A)** BMMC from WT/WT, WT/304Q, and 304Q/304Q ataxin-3 knock-in mice stimulated or unstimulated with IgE/DNP, ionomycin/PMA or LPS for 10 min. Supernatants of BMMC cultures were analyzed by Western blot for ERK1/2 phosphorylation **(A)** and IkBα **(B)** relative to GAPDH. Representative pictures and densitometric analyses as integrated density values (IDV) are shown. Unpaired t test shows statistically significant differences, n = 3. Values are mean ± SEM, *p < 0.05.

We did not observe significant differences in phosphorylation of ERK1/2 (**Figure 4A**). However, we found a significant increase of the NF-κB inhibitor IκBα in BMMC from homozygous 304Q/Q304 ataxin-3 knock-in mice (**Figure 4B**). The increase of IκBα could be responsible for a decreased expression of TNF-α as a consequence of a decreased NF-κB activity.

## Discussion

Ataxin-3, a causative protein of SCA3, is ubiquitously expressed throughout different organs, tissues and cells, including the brain and the immune system (13). Since the discovery of the polyQ-expanded ataxin-3 in patients with SCA3 (1) it was reported that this protein causes a complex pattern of gene expression changes in transgenic SCA3 cell lines and human SCA3 pontine neurons (14). Various molecular mechanisms have been proposed to explain the toxic function of polyQ expansion, including dysregulation of transcription, impairment of the ubiquitin–proteasome system, mitochondrial dysfunction, dysregulation of intracellular Ca2^+^ homeostasis, impairment of axonal transport and genotoxic stress (15). These single-cause hypotheses are not entirely satisfactory and, taken together, indicate that the disease pathogenesis might not be exclusive to one particular structure or dysfunction. Therefore, further studies are required to evaluate the implications of a polyQ expansion in SCA3 toxicity.

Mast cells were recently implicated as agents of neurodegeneration in several disease (16). Mast cells are potent activators and regulators of the peripheral immune system with access to neuronal tissue. They are able to affect the blood-brain barrier and thus could be involved in CNS pathology. Mast cells are involved in a multitude of conditions including cancer, allergy, psoriasis, multiple sclerosis, Parkinson’s disease, stroke, autism, migraines, sleep disorders, stress and inflammation (17). Brain mast cells are crucial mediators of sleep and fundamental neurobehavior which are all involved in ataxia and similar neurodegenerative disorders (18).

In this study we evaluated the hypothesis of mast cell contribution to SCA3. For this purpose, we tested the mRNA and protein expression levels of the inflammatory mast cell marker CD117/c-Kit and IL-3Rα in the brains of normal and 304Q ataxin-3 knock-in mice. CD117/c-Kit signaling network plays an important role in several cell functions including proliferation, survival, apoptosis, motility, adhesion and angiogenesis (19). We found that the expression of CD117, but not of IL-3Rα, was reduced in comparison with healthy controls. In order to analyze the effect of expanded ataxin-3 directly in mast cells we established the BMMC cultures from homozygous and heterozygous 304Q ataxin-3 knock-in mice. In agreement with the brain data, expression levels of CD117 in BMMC from 304Q ataxin-3 knock-in mice were reduced compared to cells from WT/WT mice in stimulated and unstimulated cells. We conclude that mast cells are negatively regulated by the expanded ataxin-3. Analyses of post-mortem brain samples from the SCA3 patients corroborate this conclusion.

Consistently, we found that maturation of BMMC from 304Q/304Q ataxin-3 knock-in mice takes longer than from homozygous WT and heterozygous 304Q ataxin-3 knock-in mice. However, these findings need to be confirmed in future studies by more quantitative techniques such as flow cytometry or transmission electron microscopy. Furthermore, the cell recovery/proliferation of pure mature BMMC from 304Q/304Q ataxin-3 knock-in mice was lower compared to BMMC from WT controls. Interestingly, expression of mutant huntingtin, the disease protein in Huntington´s disease (HD), was also shown to affect maturation of BMMC (20). This finding combined with our observation suggests a significant impairment in mast cells maturation in polyQ-associated neurodegenerative conditions such as SCA3 and HD.

In general, mast cells exert their effects *via* expression and release of mediators involved in physiological functions of the body through the intracellular activation of tyrosine kinases (5). The best-known activation of mast cells occurs during type-I allergic reactions through crosslinking of IgE bound to FcϵRI receptors by antigens (6). However, no effect of 304Q ataxin-3 on degranulation was found in our experiments with BMMC judging by the amounts of released β-hexosaminidase following stimulation with IgE/DNP or ionomycin/PMA. We conclude that the mast cell signaling involved in degranulation is not targeted by the expanded ataxin-3.

Along with the reduced expression of CD117, we detected a suppressive effect of expanded ataxin-3 on the mRNA and protein expression of the proinflammatory TNF-α both in the cerebellar brain regions and supernatants of ionomycin/PMA-stimulated BMMC. TNF-α expression in response to FcϵRI cross-linking was low compared to ionomycin/PMA stimulation. This may be due to different time courses and the strength of the ionomycin/PMA stimulation which skips receptor-mediated signals and directly induces PKC phosphorylation and calcium concentration elevation, respectively (21). TNF-α is a pleiotropic cytokine. Activation of signaling by TNF-α initiates a variety of potential outcomes, including cell proliferation, gene activation or cell death (22). The intracellular signaling pathways induced by this cytokine in SCA3 along with the role of ataxin-3 are only beginning to be examined (8).

In contrast to CD117 and TNF-α, the mRNA and protein levels of CXCL1 were found stimulated by the expanded ataxin-3 both in the brains and BMMC. Mast cells, like macrophages, have the capacity to newly synthesize CXCL1, making detectable amounts within 1 hour of LPS treatment (23). CXCL1 is a mouse homologue to human IL-8/CXCL8 involved in a number of pro-inflammatory activities, including the chemotaxis of neutrophils to the site of injury thereby aggravating the ongoing inflammatory response. CXCL1 is also shown to display a neuroprotective function (24). Future work will reveal the specific role for the mast cell-released CXCL1 stimulation in response to expanded ataxin-3 in the mechanism of SCA3.

The modulation of transcription of TNF-α and CXCL1 in BMMC expressing the expanded 304Q ataxin-3 compared to the healthy controls prompted us to test if this process involves NF-κB which is a protein complex that controls transcription, cytokine/chemokine production and cell survival. NF-κB is found in almost all animal cell types and is involved in cellular responses to diverse stimuli. Canonically, TNF-α is one of the major inducers of NF-κB activity (25) which in turn is expected to activate expression of CXCL1 (26). Therefore, the reduced expression of TNF-α in the BMMC from 304Q ataxin-3 knock-in mice is consistent with the increased expression of the NF-κB inhibitor, IκBα, but not with the increased expression of CXCL1 we observed. Moreover, CXCL1 binding to its receptors CXCR1/2 is supposed to activate the downstream extracellular signal-regulated kinases 1 and 2 (ERK1/2) (27). However, we did not observe significant differences in phosphorylation of ERK1/2. This conundrum points out to the profound interference of the polyQ-expanded ataxin-3 with the normal transcriptional and signaling responses of mast cells. The exploration of alternative pathways is warranted to clarify this point further.

Ataxin-3 is a deubiquitylating enzyme that removes and disassembles ubiquitin chains from specific substrates (28). NF-κB signaling consists of a series of positive and negative regulatory elements. Inducing stimuli trigger IKK activation leading to phosphorylation, ubiquitination, and degradation of IκB proteins, resulting in release of NF-κB, its nuclear translocation and ultimately induction of NF-κB target genes (29). However, a direct interaction of NF-κB pathway and the deubiquitinase ataxin-3 has not yet been shown. A possible pathway that activates NF-κB signaling in context of expanded ataxin-3 could be *via* the U-box E3 ligase CHIP, which has been identified as a direct interaction partner of ataxin-3. Thereby, ataxin-3 and CHIP interact and regulate each other’s activity (28). On the other side, CHIP was shown to promote the activation of NF-κB signaling (30). Additionally, an siRNA-based drug screen in mammalian cells expressing expanded ataxin-3 identified 15 genes which are related to TNF/NF-κB and ERK1/2 pathways and concluded that expanded ataxin-3 can be regulated by these pro-inflammatory and cell death/survival pathways (31). Moreover, ataxin-3 may be involved in deubiquitination of several signaling molecules and it is hard to predict how expanded ataxin-3 may affect certain modifications and thus the behavior or fate of such proteins.

Recently, expression of mutant huntingtin (mHtt) was shown to affect toll-like receptor 4 (TLR-4) intracellular trafficking and cytokine production in BMMC (20). Similar to our findings in BMMC with the expanded ataxin-3, expression of TNF-α was reduced while the release of β-hexosaminidase was not affected in mHtt-expressing BMMC. Besides NF-κB several transcription factors such as AP-1, SP1, ETS-1, ELK-1, and NFAT have been found to be involved in TNF-α mRNA production (32). Pérez-Rodríguez et al. report that the ERK-ELK-c-Fos signaling axis, but not NF-κB, participate in TNF-α gene transcription in BMMCs in response to LPS stimulation *via* TLR-4. We found the highest induction of TNF-α in response to stimulation with ionomycin/PMA, which is known to cause PKC activation and an increase in intracellular calcium (21). Consistently, we found a strong activation of the downstream signaling molecules ERK1/2 in response to ionomycin/PMA stimulation. Thus, TNF-α expression in response to ionomycin/PMA also appears to depend on ERK1/2-related transcription factors such as ELK-1. However, different from the findings in BMMC expressing mutant huntingtin, we observed no significant differences in the phosphorylation of ERK1/2 in BMMC with expanded ataxin-3 compared to wild type BMMC. Further investigations are needed to identify all signaling molecules involved and all effects of ataxin-3 in mast cells.

In summary, we identified for the first time the mast cell target genes transcriptionally dysregulated by the polyQ-expanded ataxin-3. Future work will reveal the pathogenic mechanisms behind these observations and will examine the previous hypothesis that transcriptional dysregulation appears to represent a unifying feature of polyQ disorders (14).

## Data Availability Statement

The original contributions presented in the study are included in the article/**Supplementary Material**. Further inquiries can be directed to the corresponding authors.

## Ethics Statement

The studies involving human participants were reviewed and approved by Ethics Committee Tübingen. The patients/participants provided their written informed consent to participate in this study. The animal study was reviewed and approved by Institutional Animal Care and Use Committee (IACUC) of the University of Tübingen.

## Author Contributions

AS and AL contributed to conception and design of the study. AS, EH, JH-S, and AL performed experiments and data analyses. AS wrote the first draft of the manuscript. All authors contributed to manuscript revision, read, and approved the submitted version.

## Conflict of Interest

The authors declare that the research was conducted in the absence of any commercial or financial relationships that could be construed as a potential conflict of interest.

## Publisher’s Note

All claims expressed in this article are solely those of the authors and do not necessarily represent those of their affiliated organizations, or those of the publisher, the editors and the reviewers. Any product that may be evaluated in this article, or claim that may be made by its manufacturer, is not guaranteed or endorsed by the publisher.

## Abbreviations

ATXN3, ataxin-3 gene/protein; BMMC, bone marrow-derived mast cells; CD117, proto-oncogene c-KIT; ERK, extracellular signal-regulated kinases; LPS, lipopolysaccharide; NF-κB, nuclear factor kappa B; PMA, Phorbol-12-myristat-13-acetat; SCA3, spinocerebellar ataxia type 3; MJD, Machado Joseph Disease; TNF-α, tumor necrosis factor alpha; IL, interleukin; DNP, 2,4-dinitrophenyl; GAPDH, glyceraldehyde 3-phosphate dehydrogenase; CXCL, C-X-C motif chemokine ligand; WT, wild type.
