# Supplementary figures and images for: Ataxin-3, The Spinocerebellar Ataxia Type 3 Neurodegenerative Disorder Protein, Affects Mast Cell Functions
Source: Front Immunol. 2022 Apr 26;13:870966. doi: 10.3389/fimmu.2022.870966 (PMC9086395; doi:10.3389/fimmu.2022.870966)

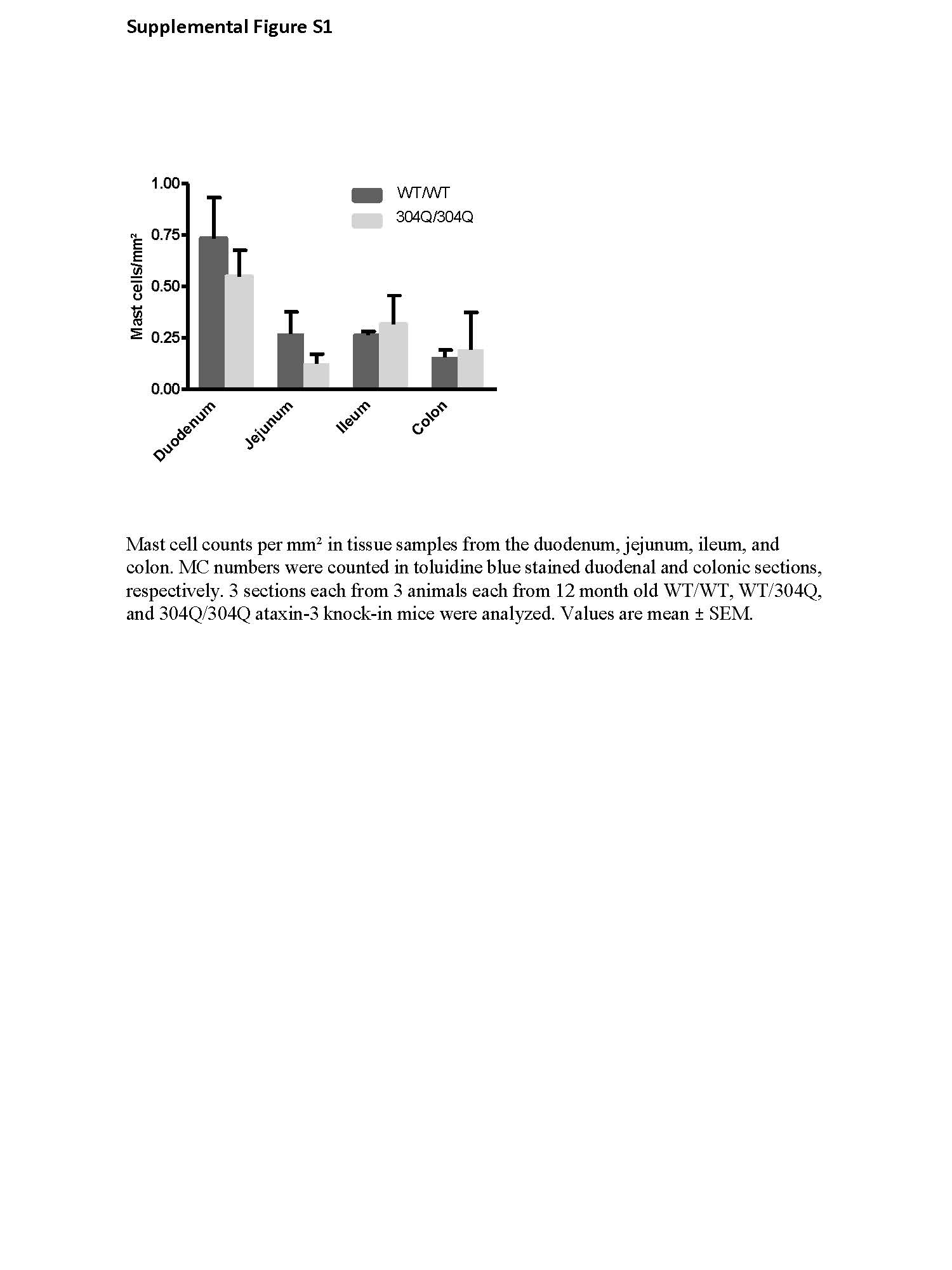

Supplement: Supplementary file 1 [file Image_1.jpeg]
